# Interleukin-7 receptor-*α* gene mutations are not detected in adult T-cell acute lymphoblastic leukemia

**DOI:** 10.1002/cam4.194

**Published:** 2014-03-26

**Authors:** Uri Rozovski, Ping Li, David Harris, Maro Ohanian, Hagop Kantarjian, Zeev Estrov

**Affiliations:** Department of Leukemia, The University of Texas MD Anderson Cancer CenterHouston, Texas

**Keywords:** Cancer genetics, hematalogical cancer, IL7rR mutations, T-ALL

## Abstract

Somatic mutations in cancer cell genes are classified according to their functional significance. Those that provide the malignant cells with significant advantage are collectively referred to as driver mutations and those that do not, are the passenger mutations. Accordingly, analytical criteria to distinguish driver mutations from passenger mutations have been recently suggested. Recent studies revealed mutations in interleukin-7 receptor-*α* (IL7R) gene in 10% of pediatric T-cell acute lymphoblastic leukemia (T-ALL) patients and in only a few cases of pediatric B-ALL. *IL7R* mutations are also frequently found in patients with lung cancer, but whereas in pediatric T-ALL *IL7R* mutations are “drivers” (consisting of gain-of-function mutations within a narrow 50-base pair interval at exon 6 that confer cytokine-independent cell growth and promote tumor transformation), in lung cancer, mutations are substitution mutations randomly distributed across the gene and are probably only “passenger” events. Because the treatment response of adult T-ALL is significantly poorer than that of childhood T-ALL and because exon 6 *IL7R* mutations play a role in the pathogenesis of childhood T-ALL, we sought to determine how the pattern of *IL7R* mutations varies between adult and childhood T-ALL. To that end, we sequenced the 50-base pair interval in exon 6 of the IL7R of DNA obtained from bone marrow samples of 35 randomly selected adult patients with T-ALL. Our analysis revealed that none of these 35 samples carried an *IL7R* mutation in exon 6. Whether differences in the genetic makeup of adult and childhood T-ALL explain the differential response to therapy remains to be determined.

## Introduction

T-cell acute lymphoblastic leukemia (T-ALL), which accounts for approximately 15% of childhood ALL and 25% of adult ALL cases 1, is characterized by a profound infiltration of the bone marrow with clonally rearranged T-cell receptor (TCR) 2 lymphoblasts 3 that stain positively for TdT and express CD1a, CD2, CD3, CD5, CD7, and CD8 1. An abnormal karyotype is usually detected in the lymphoblasts of 50–70% of T-ALL patients. The most common abnormalities are translocations causing the deregulation of transcription factors that are juxtaposed with a regulatory region of one of the TCR loci 1. Currently, nearly 80% of pediatric T-ALL cases are cured with intensive chemotherapy 4. However, adults with T-ALL fare much worse, and only approximately 50% survive for 5 years after being diagnosed 5.

In T-ALL, as in most other neoplasms, the neoplastic cell genome harbors somatic mutations. The number of somatic mutations in cancer cells ranges from less than 10 in neoplasms such as childhood ALL to more than 200 in melanoma or lung cancer 6. Mutations in cancer cell genes are broadly categorized as those that provide malignant cells with a proliferative or survival advantage, collectively termed “driver” mutations, or those that do not, which are therefore termed “passenger” mutations. Similar terminology is used for genes: A driver gene is a mutated gene that provides the cell with a significant growth advantage 6. The identification of driver genes is relatively easy if the frequency of mutations in a given gene is very high, as is the case for *TP53* in colon cancers 7. In other cases, identifying driver mutations or driver genes might be challenging. Presumably, when an oncogene is recurrently mutated at the same amino acid position, this signifies a driver mutation, and driver mutations in tumor suppressor genes are those that cause protein truncation alterations 6.

Several somatic mutations have been recurrently identified in T-ALL including activating mutations of the *NOTCH1* gene, which encode a protein that is critical for early T-cell development 8, or in the *FBXW7* gene, a negative regulator of NOTCH1 9. Mutations in *FBXW7*, *PHF6*, *DNMT3A*, *RUNX1*, *JAK1*, and *WT1* as well as deletions of the *CDK2A/B* tumor suppressor genes have also been reported 10.

The cell surface interleukin (IL)-7 receptor-*α* (IL7R), present in lymphoid progenitor cells, is required for normal lymphocyte development 11. IL7R forms heterodimers with IL-2R*γ* or with the cytokine receptor-like factor 2 (CRLF2) 12 and activates the JAK/STAT5 and the PI3K/Akt/mTOR signaling pathways 13. *IL7R* mutations have been identified in malignant and nonmalignant diseases. Germ line loss-of-function mutations were found to be associated with the development of severe combined immunodeficiency 14. Somatic mutations in *IL7R* have been identified in lung cancer patients where its functional significance is unknown, and mutations in IL7R are detected in 10% of pediatric T-ALL cases and in a few cases of pediatric B-ALL.

Because the distribution of mutations across a gene provides indications for its functional significance, we analyzed the distribution pattern of *IL7R* in pediatric ALL and lung cancer patients. Subsequently, we screened bone marrow samples of 35 adult patients with T-ALL for exon 6-*IL7R* mutations and did not detect a single case with mutations in exon6-*IL7R*. Our findings suggest that exon 6 *IL7R* mutations are driver events in childhood T-ALL that are not shared by adult T-ALL patients.

## Materials and Methods

### Patients

Bone marrow cells were obtained from 35 patients 18 years or older with T-ALL treated at The University of Texas MD Anderson Cancer Center leukemia clinic from 2002 to 2010. Institutional Review Board approval and patients' written informed consent were obtained. The clinical characteristics of the patients whose bone marrow samples were used in this study are presented in Table 1.

**Table 1:** Characteristics of the T-ALL pediatric cohort patients

*N*	35
Age	
Median (range)	32.5 (18–76)
Gender	
Males *N* (%)	25 (76%)
Females *N* (%)	8 (24%)
Karyotype	
Normal	16 (52%)
Aberrant	15 (48%)
No data available	4
White blood cell count (×10^9^/L)	
Median (range)	8.7 (0.6–106)
Platelet count (×10^9^/L)	
Median (range)	119 (11–612)
Hemoglobin levels (g/L)	
Median (range)	10.6 (4.4–15.2)

### Molecular analysis

To isolate low-density cells, bone marrow cells were fractionated using Histopaque 1077 (Sigma–Aldrich, St. Louis, MO). Genomic DNA was isolated by using a QIAamp DNA Blood Maxi Kit (Qiagen, Valencia, CA) according to the manufacturer's instructions. Briefly, bone marrow low-density cells were washed twice with cold phosphate-buffered saline, and 2 × 10^7^ cells were resuspended in 5 mL of phosphate-buffered saline. Cells were lysated with 12 mL of Buffer AL and digested with 500 *μ*L Qiagen Protease.

After the lysates were incubated for 10 min at 70°C, 10 mL of ethanol was added and mixed by inverting the tube several times. All of the lysates were loaded onto the QIAamp Maxi columns. The columns were washed with buffer AW1 and AW2. The genomic DNA was eluted with nuclease-free water, and 10–50 ng of genomic DNA was analyzed using polymerase chain reaction (PCR). PCR was performed by using a TaKaRa Hot Start PCR kit. The following primers directed at exon 6 of *IL7R* were used: forward: caactttcaggaaataataagtgg; reverse: taaattcgtgaaatgccttaatcc. The PCR products were run on an agarose gel, excised, purified with a QIAquick PCR purification kit (Qiagen), and sequenced. Sequencing of those purified PCR products was performed by SeqWright (Houston, TX).

## Results and Discussion

### Different *IL7R-α* mutations induce dissimilar functions

The catalog of somatic mutations in cancer (COSMIC) database (http://cancer.sanger.ac.uk/cancergenome/projects/cosmic/) contains 127 *IL7R* somatic mutations. Most mutations are associated with the transcription of a full-length protein product including amino acid substitution mutations (32%), in-frame insertions (24%), and complex mutations (33%). None of the recurrent *IL7R* mutations are protein-truncating. *IL7R* mutations were found in squamous cell carcinoma (12/179; 6.7%) and adenocarcinoma (15/474; 3.2%) of the lung, pediatric T-ALL (64/665; 9.6%), B-ALL (13/276; 4.7%), and sporadically in other tumors. Remarkably, for each tissue, the type of mutations and the distribution across the *IL7R-α* gene are different. In lung tumors, substitution mutations are distributed across the entire *IL7R* gene in a seemingly random fashion. In childhood T-ALL, all *IL7R* mutations occur within a narrow 50-base pair interval at exon 6, encompassing in-frame insertions or deletion/insertion mutations.

**Figure 1:**
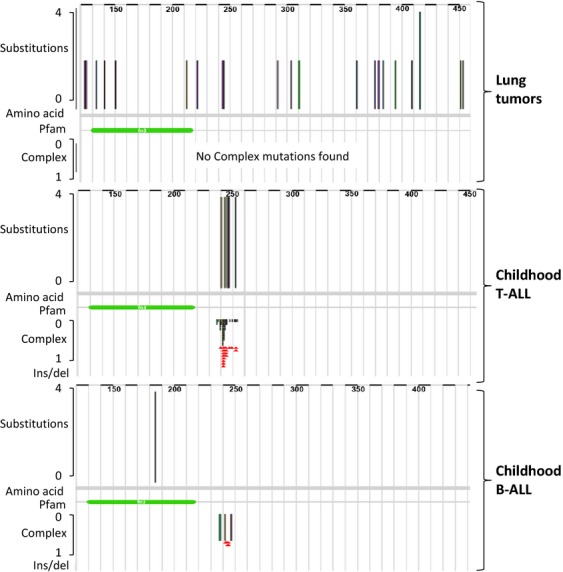
Distribution of somatic *IL7R* mutations in different tumors. All mutations in *IL7R* are in-frame mutations, resulting in the translation of a full-length protein product. Upper panel: in lung tumors, substitution mutations are dispread across the entire gene appear usually only once and carry no functional significance. Middle panel: all mutations in childhood T-ALL are within the exon 6 narrow region of 50 base pairs. These mutations induce the production of a protein that is an oncogenic driver. Lower panel: in addition to mutations in exon 6, childhood B-ALL is characterized by serine-to-cysteine substitution in the extracellular domain, consistent with two driver oncogenic events, one common to both childhood B-ALL and T-ALL and one unique to childhood B-ALL. The figure was generated using the COSMIC database (http://cancer.sanger.ac.uk/cancergenome/projects/cosmic/). COSMIC, catalog of somatic mutations in cancer.

The random distribution pattern of *IL7R* mutations across the IL7R gene in lung tumors versus the nonrandom distribution in pediatric ALL suggests a fundamental difference in the significance of *IL7R* mutation between the two entities: in tumors with a high mutation rate (e.g., lung tumor) there is a high probability of finding passenger, randomly inserted mutations. These are usually nonrecurrent, substitution mutations that are randomly spread across the gene. Conversely, when mutations are identified primarily in one or few intragenic sites, they probably confer a functional significance (Fig. 1). Indeed, exon 6 *IL7R* mutations in pediatric T-ALL cases conferred cytokine-independent cell growth and promoted tumor cell transformation, confirming their functional significance as a “driver,” gain-of-function mutation.

Two types of *IL7R* mutations have been identified in childhood ALL. The first type includes mutations affecting the transmembrane domain, whereby disulfide homodimerization of two mutant receptors induces constitutive activation of downstream signaling pathways, usually induced only upon ligand binding to the heterodimer receptor complex. This mutation is found in both childhood T-ALL and B-ALL. The second type includes a recurrent serine-to-cysteine substitution mutation at amino acid 185 in the extracellular *IL7R* domain. This mutation has been detected exclusively in B-ALL and almost always in association with mutations in the *CRLF2* gene 15, thereby suggesting that these mutations provide an advantage to B-lineage commitment, perhaps by inducing a weaker signal that preferentially activates only one signaling pathway. The *IL7R* insertion mutations at exon 6 were detected in both childhood T-ALL and B-ALL, whereas a recurrent serine-to-cysteine substitution at amino acid 185 (the S185C mutation in the *IL7R* extracellular domain) was identified exclusively in childhood B-ALL.

### *IL7R* mutations are not detected in adult T-ALL

Although activating mutations of *NOTCH1* have been detected in 70% of both pediatric and adult T-ALL cases 3,10, significant age-related differences in the frequencies of other genetic alterations have been reported. For example, t(7;10)(q34;q24) and t(10;14)(q24;q11), both of which involve the translocation of the TCR in juxtaposition to the HOX11 transcription factor, occur in 7% of childhood T-ALL cases and 30% of adult T-ALL cases 16. Similarly, a higher prevalence of *FBXW7* and *PHF6* mutations was reported in adult T-ALL 10,17,18.

Because the *IL7R* in-frame insertion mutation in exon 6 is a gain-of-function oncogenic driver mutation found in 10% of childhood T-ALL cases, we sought to determine whether this mutation is also present in adult T-ALL. A power calculation showed that analysis of 35 T-ALL samples has a power of 0.8 to detect an *IL7R* mutation if present in 10% or more of the samples. Therefore, we analyzed bone marrow aspirates from 35 adult patients with T-ALL for the presence of *IL7R* mutations at exon 6. *IL7R* insertion mutation was not detected in any of these cases. Whether age-dependent differences in the genetic makeup of T-ALL (signified by the lack of *IL7R* mutations) explain differences in response to therapy between adult and childhood T-ALL remains to be determined.

## References

[1] Swerdlow SH, Campo E, Harris NL, Jaffe ES, Pileri SA, Stein H (2008). WHO Classification of tumours of Haematopoietic and Lymphoid tissuesed.

[2] Pilozzi E, Muller-Hermelink HK, Falini B, de Wolf-Peeters C, Fidler C, Gatter K (1999). Gene rearrangements in T-cell lymphoblastic lymphoma. J. Pathol.

[3] Ferrando AA (2009). The role of NOTCH1 signaling in T-ALL. Hematology Am. Soc. Hematol. Educ. Program.

[4] Goldberg JM, Silverman LB, Levy DE, Dalton VK, Gelber RD, Lehmann L (2003). Childhood T-cell acute lymphoblastic leukemia: the Dana-Farber Cancer Institute acute lymphoblastic leukemia consortium experience. J. Clin. Oncol.

[5] Marks DI, Paietta EM, Moorman AV, Richards SM, Buck G, DeWald G (2009). T-cell acute lymphoblastic leukemia in adults: clinical features, immunophenotype, cytogenetics, and outcome from the large randomized prospective trial (UKALL XII/ECOG 2993). Blood.

[6] Vogelstein B, Papadopoulos N, Velculescu VE, Zhou S, Diaz LA (2013). Kinzler KW. Cancer genome landscapes. Science.

[7] Iacopetta B (2003). TP53 mutation in colorectal cancer. Hum. Mutat.

[8] Weng AP, Ferrando AA, Lee W, Morris JPT, Silverman LB, Sanchez-Irizarry C (2004). Activating mutations of NOTCH1 in human T cell acute lymphoblastic leukemia. Science.

[9] Malyukova A, Dohda T, von der Lehr N, Akhoondi S, Corcoran M, Heyman M (2007). The tumor suppressor gene hCDC4 is frequently mutated in human T-cell acute lymphoblastic leukemia with functional consequences for Notch signaling. Cancer Res.

[10] Grossmann V, Haferlach C, Weissmann S, Roller A, Schindela S, Poetzinger F (2013). The molecular profile of adult T-cell acute lymphoblastic leukemia: mutations in RUNX1 and DNMT3A are associated with poor prognosis in T-ALL. Genes Chromosom. Cancer.

[11] Peschon JJ, Morrissey PJ, Grabstein KH, Ramsdell FJ, Maraskovsky E, Gliniak BC (1994). Early lymphocyte expansion is severely impaired in interleukin 7 receptor-deficient mice. J. Exp. Med.

[12] Noguchi M, Nakamura Y, Russell SM, Ziegler SF, Tsang M, Cao X (1993). Interleukin-2 receptor gamma chain: a functional component of the interleukin-7 receptor. Science.

[13] Ribeiro D, Melao A, Barata JT (2013). IL-7R-mediated signaling in T-cell acute lymphoblastic leukemia. Adv. Biol. Regul.

[14] Roifman CM, Zhang J, Chitayat D, Sharfe N (2000). A partial deficiency of interleukin-7R alpha is sufficient to abrogate T-cell development and cause severe combined immunodeficiency. Blood.

[15] Shochat C, Tal N, Bandapalli OR, Palmi C, Ganmore I, te Kronnie G (2011). Gain-of-function mutations in interleukin-7 receptor-alpha (IL7R) in childhood acute lymphoblastic leukemias. J. Exp. Med.

[16] Graux C, Cools J, Michaux L, Vandenberghe P, Hagemeijer A (2006). Cytogenetics and molecular genetics of T-cell acute lymphoblastic leukemia: from thymocyte to lymphoblast. Leukemia.

[17] Kraszewska MD, Dawidowska M, Kosmalska M, Sedek L, Grzeszczak W, Kowalczyk JR (2013). BCL11B, FLT3, NOTCH1 and FBXW7 mutation status in T-cell acute lymphoblastic leukemia patients. Blood Cells Mol. Dis.

[18] Van Vlierberghe P, Palomero T, Khiabanian H, Van der Meulen J, Castillo M, Van Roy N (2010). PHF6 mutations in T-cell acute lymphoblastic leukemia. Nat. Genet.

